# The Biological Effect of Enriching the Plasma Content in Platelet-Rich Plasma: An In Vitro Study

**DOI:** 10.3390/biom14101328

**Published:** 2024-10-18

**Authors:** Eduardo Anitua, Mar Zalduendo, Roberto Prado, María Troya, Roberto Tierno, María de la Fuente, Mohammad H. Alkhraisat

**Affiliations:** 1 University Institute for Regenerative Medicine and Oral Implantology, UIRMI (UPV/EHU-Fundación Eduardo Anitua), 01007 Vitoria, Spain; marimar.zalduendo@bti-implant.es (M.Z.); roberto.prado@bti-implant.es (R.P.); maria.troya@bti-implant.es (M.T.); roberto.tierno@bti-implant.es (R.T.); maria.delafuente@bti-implant.es (M.d.l.F.); mohammad.hamdan@bti-implant.es (M.H.A.); 2 BTI-Biotechnology Institute, 01005 Vitoria, Spain

**Keywords:** cell therapy, lyophilization, plasma content enrichment, platelet-rich plasma, tissue regeneration

## Abstract

BACKGROUND: Platelet-rich plasma (PRP) formulations have become valuable therapeutic tools in regenerative medicine. In addition, these blood derivates have been successfully included in cell therapy as fetal bovine serum substitutes, due to the real need to avoid the risk of host immunologic reactions and the animal disease transmission associated with reagents from animal origin. However, the protocols for obtaining them should be optimized to improve their biological potential. METHODS: PRP-derived preparations with different concentrations of the platelet and plasma components were obtained from the blood of five donors by freeze-drying. Measurements of the pH, protein, and growth factor concentration were performed. Moreover, their biological effects on cell proliferation and migration and their angiogenic potential were assessed. RESULTS: An increased plasma component concentration resulted in an augmented quantity of the total protein content, a significative variation in the hepatocyte growth factor concentration, and an experimental but clinically irrelevant alteration of the pH value. No significant changes were induced in their potential to enhance proliferative and migratory responses in epithelial cells, with the latter being reduced for dermal fibroblasts. The endothelial cell capacity for tube formation was significatively reduced. CONCLUSIONS: An increased blood plasma content did not improve the biological potential of the formulations. However, they have emerged as a promising approach for regenerative therapies where neovascularization must be avoided.

## 1. Introduction

Many autologous or allogeneic human blood-derived products, mainly human serum, platelet-rich plasma (PRP), or platelet lysates, have been proposed as potential FBS replacements in cell therapy [1,2,3,4,5,6], in order to avoid the risk of possible host immunologic reactions, as well as the transmission of animal diseases. Furthermore, since the 1990s, PRP has been progressively applied in numerous medical fields, because of the healing potential for tissue regeneration of all its derivates extensively reported in dentistry, orthopedics, ophthalmology, dermatology, gynecology, urology, cardiology, and veterinary, among others areas of medicine [7,8,9,10]. One of these PRP derivates is the supernatant obtained after coagulation, which contains circulating molecules in addition to the platelet secretoma. The therapeutic potential of this formulation has been confirmed both for the treatment of ocular disorders when applied as eye drops [11,12] and in cell therapies for the safe and effective ex vivo maintenance of isolated cells, when utilized as a culture medium supplement [13].

Platelets store biomolecules capable of triggering cell proliferation, migration, or differentiation pathways; promoting tissue repair; and regulating angiogenesis, inflammation, and the immune response [14,15,16,17]. Potent growth factors, such as platelet-derived growth factor (PDGF), beta-transforming growth factor (β-TGF), epidermal growth factor (EGF), basic fibroblastic growth factor (b-FGF), angiopoietin-2, and vascular endothelial growth factor (VEGF), among other bioactive modulators, have been identified as associated with the platelet secretoma [18]. In addition, platelets contain preexisting mRNAs, as well as noncoding RNAs; display specialized mechanisms for processing RNA transcripts; and conduct post-translational modifications of the proteins, which may alter the constitutive platelet proteome in response to activating signals [19,20,21]. Moreover, platelets contain the cytokines and bioactive lipid products—of particular relevance among which are the eicosanoids, which are capable of secreting exosomes and microparticles—involved in cell-to-cell communication upon activation and as a result of other processes, such as the shear forces generated by flowing blood [22,23,24,25,26].

In an attempt to optimize the final therapeutic product and due to the great relevance of the platelet secretoma, the protocols for obtaining PRP formulations have undergone continuous modifications focused on obtaining plasma formulations highly enriched in platelets [27,28,29,30,31,32]. However, the very high complexity of the human blood–plasma matrix containing the platelet-derived molecules should not be ignored. In addition to electrolytes and respiratory gases, a multitude of proteins and low-molecular weight lipid biomolecules have been determined through different technologies [33,34]. Traditionally, the functions of buffering blood pH and maintaining osmotic balance, as well as the role of being a carrier for the transport of other molecules and the key agents in blood coagulation, have been extensively attributed to the solid component of plasma. Nevertheless, the functions as modulators of several cell activities involved in the initiation and resolution of the inflammation processes have recently been attributed to the bioactive lipids of the human plasma [35,36]. In addition, a potential role for skeletal muscle healing and regeneration has been reported for n-3 PUFAs (polyunsatured fatty acids) and their lipid mediators [37]. On the other hand, in vitro-induced modifications in the lipidic composition of the cell membrane have been proposed as the regulators of mesenchymal stem cell (MSC) differentiation [38]. Moreover, research on the extracellular vesicles (EVs) circulating in the blood gained interest at the beginning of the present century, due to their discovery as a new communication system between cells. The EVs generated not only by hematopoietic cells but also by adipocyte, hepatocyte, muscle, and endothelial cells, among others, can reach most of the tissues of the organism, even crossing the blood–brain barrier [39,40,41,42,43,44,45,46]. Other plasma molecules such as stable free forms of circulating noncoding RNAs, in addition to those found within the membrane structures, could also control the gene expression and differentiation in recipient cells [47,48]. In this regard, a differential distribution of RNA species and functional RNA transcripts in different human plasma formulations has been reported [49]. In addition, circulating DNA, associated with small EVs, exosomes, or protein complexes, as well as within extracellular mitochondria, has been postulated to be another novel mediator for cell–cell communications [50,51,52].

Considering the extreme complexity of the blood plasma component in PRP, we decided to assess its possible involvement in the triggering of cellular responses that could improve regeneration. Thus, the aim of this research was to design and obtain blood-derived formulations with different concentrations of plasma and platelet contents. Moreover, their biological effect on two cell phenotypes was determined, as well as their contribution to the promotion of angiogenesis.

## 2. Materials and Methods

The study was performed in accordance with the ethical standards from the Araba University Hospital Clinical Research Ethical Committee (BTI-01-IV/22/CPL), approved on 7 November 2022, and following the principles established in the Declaration of Helsinki amended in 2013.

### 2.1. The Obtaining and Hematological Characterization of Blood-Derived PRP Formulations

The different plasma formulations were identified with a code consisting of 2 numbers separated by a dash, where the first one referred to the plasmatic factor content and the second one to the platelet concentration, with both parameters being compared to the peripheral blood.

**Figure 1:**
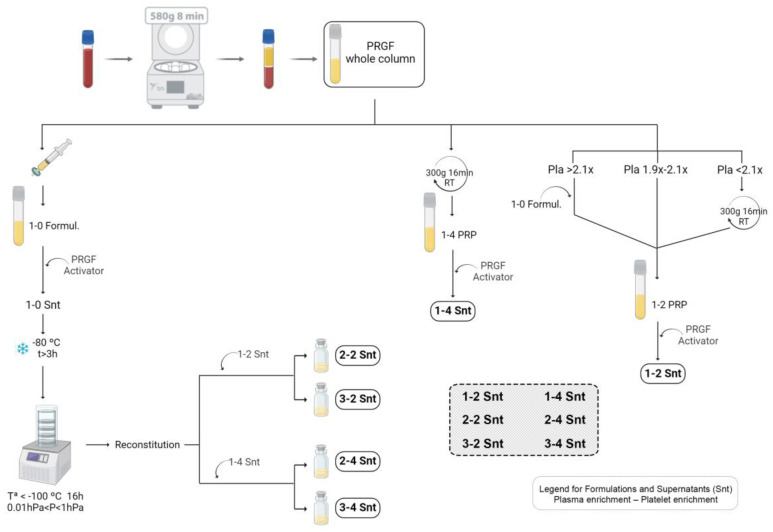
Schematic illustration showing the process for obtaining the 6 supernatant preparations prepared from plasma rich in growth factors (PRGF). PRGF: Plasma Rich in Growth Factors. 1-0 formulation: preparation with 1× plasma content and no platelets. 1-2 PRP: Plasma Rich in Platelets with 1× and 2× concentration factors in plasma and platelet contents, respectively. 1-4 PRP: Plasma Rich in Platelets with 1× and 4× concentration factors in plasma and platelet contents, respectively. Snt: supernatant for cell culture medium supplementation. 1-2 Snt: supernatant with 1× and 2× plasma and platelet content, respectively. 2-2 Snt: supernatant with 2× plasma and platelet content. 3-2 Snt: supernatant with 3× and 2× plasma and platelet content, respectively. 1-4 Snt: supernatant with 1× and 4× plasma and platelet content, respectively. 2-4 Snt: supernatant with 2× and 4× plasma and platelet content, respectively. 3-4 Snt: supernatant with 3× and 4× plasma and platelet content, respectively. Created with BioRender.com.

Blood from 5 healthy donors ranging in age from 25 to 51 years old (3 male and 2 female) was collected into tubes with 3.8% (*wt/v*) sodium citrate, after the signing of a written informed consent. The blood was centrifuged at 580 *g* for 8 min at RT (Endoret System; BTI Biotechnology Institute, S.L., Miñano, Spain). The whole plasma column was collected to obtain plasma rich in growth factors (PRGF), avoiding the buffy coat. Three plasma formulations were obtained from this using different protocols. Part of the whole plasma column was filtered through a 0.22 μm sized pores, in order to obtain the platelet-free plasma preparation (1-0), which was afterwards used for the preparation of the supernatants with increased plasma content. The plasma column collected was considered a 1-2 PRP when the platelet concentration was 1.9 to 2.1 times the platelet count in the peripheral blood, while, in the case of the PRGF platelet concentration being outside this range, two different procedures were followed. Thus, for higher concentrations, the platelet count was adjusted with the required volume of 1-0 formulation. On the contrary, when the platelet concentration was lower than in the peripheral blood, the PRGF was centrifuged at 300× *g* for 16 min at RT to obtain the platelet pellet. Then, after the removal of the necessary volume, it was resuspended in the remaining supernatant itself. To obtain the third formulation, 1-4 PRP, the PRGF was centrifuged in the same conditions described just before. Then, part of the total volume of the supernatant was removed, and the pellet of platelets was suspended again in the remaining supernatant (Figure 1).

The platelet, erythrocyte, and leukocyte concentrations were determined in the 1-0 preparation and both PRP formulations (1-2 and 1-4 PRP) using a hematology analyzer (Pentra ES 60, Horiba ABX, Montpellier, France). Moreover, the mean platelet volume was also measured.

### 2.2. The Obtaining of Supernatants with Different Plasma and Platelet Contents

In order to obtain the supernatants for the culture medium supplementation, different protocols were performed. PRPs with 1-2 and 1-4 compositions were activated with the PRGF Activator (BTI Biotechnology Institute, S.L.) following the manufacturer’s instructions. Thus, 20 μL of the activator was added to each milliliter of PRP and allowed to clot at 37 °C. After 1 h, the PRP clots were centrifuged at 1000 g for 10 min at RT, and, finally, the supernatants were filtered (Figure 1). Part of these supernatants were stored into aliquots at −80 °C for the experimental assays. The supernatants were named 1-2 Snt and 1-4 Snt and contained 2× and 4× the platelet concentrations, respectively, and the same plasma content as the peripheral blood. The rest of both of the preparations was kept overnight at 4 °C for further use in the process of obtaining supernatants with a concentrated plasma content.

To prepare these samples enriched in plasma content, a 1-0 formulation was activated as described above. Then, after being filtered, the supernatant (1-0 Snt) was frozen in glass vials for lyophilization for a minimum of 3 h. Then, it was freeze-dried at a temperature below −100 °C and a pressure between 0.01 hPa and 1 hPa for 16 h (Coolsafe 110-4 Touch, Labogene Scandinavian by Design, Allerød, Denmark) (Figure 1). The lyophilized 1-0 Snt was reconstituted with the same or half of the volume of 1-2 Snt, the production of which is described in the preceding paragraph for obtaining 2-2 Snt and 3-2 Snt, with two and three times the plasma content of the peripheral blood, respectively, and both with two-fold of its platelet count. Similarly, 2-4 Snt and 3-4 Snt were prepared after adding 1-4 Snt to the lyophilized 1-0 Snt until getting the original or half the volume, respectively. The 2-4 Snt and 3-4 Snt formulations contained four times the platelet count and two and three times, respectively, the plasma content of the peripheral blood. Finally, all the samples were stored at −80 °C until use.

### 2.3. Characterization of Supernatant Preparations

The pH measurements of the 6 supernatants with different compositions were performed using a specific electrode for small sample volumes (GLP 22 Crison, HACH LANGE SPAIN, S.L.U., Hospitalet de Llobregat, Spain). The protein quantification was performed using the bicinchoninic acid (BCA) colorimetric assay based on the formation of protein–copper ion complexes, in which the absorbance is proportional to the protein concentration in the sample (Thermo Fisher Scientific, Waltham, MA, USA). Moreover, the hepatocyte growth factor (HGF), platelet-derived growth factor (PDGF-BB), and vascular endothelial growth factor (VEGF-A) were quantified in the 6 preparations by means of enzyme-linked immunosorbent assays (ELISA) (R&D Systems Inc-Biotechne, Minneapolis, MN, USA) and following the manufacturers’ instructions. The assays were performed in duplicate.

### 2.4. Cell Culture

Human dermal fibroblasts (HDFs) (ScienCell Research Laboratories, Carlsbad, CA, USA) and SV40-adeno vector-transformed human corneal epithelial cells (HCEs; Riken Bioresource Research Centre Cell Bank, Ibaraki, Japan) were used to test the biological effect of the six supernatants. Human umbilical vein endothelial cells (HUVECs) were included for the assays regarding the angiogenic process (Lonza Group Ltd., Basel, Switzerland).

The HDFs were cultured with Fibroblast Basal Medium (FM) supplemented with Fibroblast Growth Supplement (FGS), 2% FBS, and penicillin–streptomycin (all reagents provided by ScienCell Research Laboratories). The DMEM/F12 medium (Invitrogen-Gibco, Thermo Fisher Scientific), supplemented with 5 mg/mL of insulin (SigmaAldrich-Merck, Darmstadt, Germany), 10 ng/mL of EGF (Sigma-Aldrich), 1% dimethyl sulfoxide (DMSO) (Sigma-Aldrich), 7.5% FBS (Biochrom AG, Berlin, Germany), and penicillin–streptomycin (ScienCell Research Laboratories), was used for the HCE maintenance. The HUVECs were cultured in Endothelial Growth Medium (EGM), consisting of endothelial basal medium supplemented with penicillin/streptomycin and endothelial cell growth supplements (ECGSs) (all reagents purchased by Innovative Technologies in Biological System S.L., Derio, Spain), and 5% FBS (Biochrom AG). The three cell phenotypes were maintained at 5% CO_2_ at 37 °C. For the subcultures, after reaching the confluence, the cells were detached with an animal origin-free trypsin-like enzyme (TrypLE Select, Invitrogen-Gibco), and the cell viability was assessed by a trypan blue dye exclusion.

For the three cell phenotypes, the FBS supplementation was replaced by 2% supernatants in all experimental assays.

### 2.5. Cell Proliferation Assay

To test the effect on the cell proliferation of the 6 supernatants, the cells were seeded into 96-well optical-bottom black plates at a density of 5000 cells/cm^2^ for the HDFs and 18,000 cells/cm^2^ in the case of the HCEs. The treatments were added to 100 μL of the corresponding culture medium, depending on the cell phenotype, in which the FBS supplement was replaced by 2% of the six supernatant formulations obtained from each donor. The cells maintained in their specific routine culture medium were included at the optimal proliferation rate for each cell phenotype (as a positive control). Moreover, the proliferation of cells incubated with 0.1% FBS, instead of the appropriate percentage according to the cell phenotype, was associated with the initial seeding cell density (the negative control). Five replicates of each condition were analyzed. The cells were treated for 72 h, and, then, the cell proliferation was quantified using the CyQuant assay kit (Molecular Probes- Thermo Fisher Scientific) according to the manufacturer’s instructions. Briefly, the treated cells were carefully washed with a phosphate-buffered saline, after removing the culture medium, and, then, the plates were frozen at −80 °C. The thawed cells were treated with 1.35 Kunitz units/mL of RNase A (Sigma-Aldrich) in a lysis buffer for 1 h. Finally, the lysed cells were incubated with the CyQuant^®^ GR dye, which exhibits strong fluorescence enhancement when bound to cellular nucleic acids. The fluorescence was then measured directly using a fluorescence microplate reader with filters appropriate for 480 nm excitation and 520 nm emission maxima (Biotek Synergy H1 multimode reader, Agilent Technologies, Inc., Santa Clara County, CA, USA). A bacteriophage λ DNA standard curve was included to quantify the cellular DNA, provided the RNA component of the fluorescent signal was removed previously. The results for the supernatant treatments were referred to those obtained for the positive control and expressed in percentages. Five replicates were assayed for each supernatant, donor, and cell phenotype.

### 2.6. Cell Chemotaxis

The chemotactic potential of the 6 supernatants was quantified by means of a commercial 96-well fluorometric kit for the cell migration determination (CytoSelect™, Cell Biolabs, Inc., San Diego, CA, USA). Prior to the beginning of the assay, the cells were incubated for 24 h with the corresponding culture medium for each phenotype, in which the FBS supplementation was reduced to a minimum of 0.1%. This cell migration assay kit contains a polycarbonate membrane chamber (8 µm sized pores) as a barrier to discriminate migratory cells from nonmigratory cells. Migratory cells are able to pass through the pores of the polycarbonate membrane extending protrusions towards the chemoattractants.

To determine the chemotactic response of the three cell phenotypes to the six supernatants with different plasma and platelet contents, the manufacturer’s instructions were followed. Briefly, 150 μL of the appropriate culture medium, depending on the cell phenotype, supplemented with 2% PRP supernatant, instead of the corresponding percentage of FBS, was added into the feeder tray. The cells were suspended in the culture medium specific to each cell phenotype containing 0.1% FBS in substitution for its proportion in the routine culture medium. Subsequently, 100 μL of the cell suspension was seeded into the membrane chamber, with a cell density of 25,000 and 30,000 cells per well for the HDFs and HCEs, respectively. The cells were allowed to move for 24 h, when the migrated cells were harvested by dislodging in the cell detachment solution. These cells were finally incubated with Cyquant GR dye in a lysis buffer, and the DNA fluorescence was quantified at 485 nm and 535 nm excitation and emission wavelengths, respectively (Biotek Synergy H1 multimode reader, Agilent Technologies, Inc.). The chemotaxis was assayed in triplicate for each experimental condition.

### 2.7. Angiogenesis Assay: Endothelial Cell Proliferation and Tube Formation

The HUVEC proliferation was analyzed following the protocol described before. Similarly, the cells were seeded at a density of 5000 cells/cm^2^ and treated with 1-2 Snt, 2-2 Snt, and 3-2 Snt for 72 h. The same previously described positive and negative controls were included in the assay. All the experimental conditions were tested in 5 replicates. The cell proliferation was quantified using the CyQuant assay kit.

The organization of the endothelial cells into capillary-like structures in vitro was used as a sign suggestive of angiogenic potential. The tube formation assay was performed in the μ-Plate 96-well 3D system (Ibidi GmbH, Gräfelfing, Germany) previously covered with a solubilized growth factor reduced (GFR) basement membrane matrix (Matrigel^®^, Corning, Glendale, CA, USA). By means of this assay, single endothelial cells were seeded and then the tube formation over time could be observed and imaged, where the expression “tubes” describe the cords of cells that are visible in a formed network.

For this purpose, firstly, 10 µL of the matrix gel was applied to each inner well of the µ-Plate 96-well 3D, following the manufacturer’s instructions. Then, the matrix gel was allowed to polymerize for 30–60 min in the cell incubator. After this, the HUVECs were seeded in 70 μL of the culture medium at a density of 10,000 cells per well. The treatments consisted of endothelial basal medium supplemented with penicillin/streptomycin, ECGSs, and 2% supernatants of different plasma and platelet contents, replacing the 5% FBS used in the routine culture medium. Two control groups were included regarding the initial cell density (negative control: endothelial basal medium with penicillin/streptomycin, ECGSs, and 0.1% FBS) and the optimal endothelial cell growth (positive control: endothelial basal medium with penicillin/streptomycin, ECGSs, and 5% FBS). All the cells sunk to the bottom of the slide, and an homogeneous cell distribution was achieved. Three replicates were tested for each experimental and control condition. The tube formation was assessed after 20 h of incubation, then images of the central area of all the wells were taken using an inverted light microscope (Leica DM IRB, Leica Microsystems, Wetzlar Hesse, Germany) equipped with a digital camera (Leica DFC 300FX, Leica Camera AG, Wetzlar, Germany). Subsequently, the pictures were analyzed, and the angiogenic process was quantified by means of a WimTube image analysis (Wimasis GmbH, Munich, Germany). Parameters such as the covered area; the total tube length; and the total tubes, loops, and branching points were evaluated.

### 2.8. Statistical Analysis

The results were expressed as the mean ± standard deviation. A one-way repeated measures ANOVA was performed to analyze the statistically significant differences among the biological effect of the 6 supernatants derived from the blood of 5 donors. Moreover, the mean differences derived from the platelet or plasma content of the treatment and obtained for all the analyzed parameters were assessed through a two-factor repeated measures ANOVA. The Bonferroni correction was used to adjust the confidence range. A Pillai’s trace value less or equal to 0.05 was considered to assume the differences as statistically significant. All the statistical analyses were performed using SPSS software (version 23.0) (SPSS Inc., Chicago, IL, USA).

## 3. Results

### 3.1. Hematological Characterization of Blood-Derived Plasma Formulations

All the blood-derived plasma formulations required for obtaining the supernatants that would be tested as supplements for the cell culture were characterized. Thus, the erythrocyte, leukocyte, and platelet counts were performed for the 1-0, 1-2, and 1-4 formulations. No red and white blood cells were detected in the 1-0 preparation. In the case of the PRP formulations, the counts were absent and negligible for the erythrocyte and leukocyte cells, respectively, as can be seen in Table 1.

Moreover, 1.9-fold and 3.3-fold of the platelet concentrations of the peripheral blood were obtained for the 1-2 and 1-4 formulations, respectively, while no platelets were found in the 1-0 preparation. A low value was obtained for the platelet concentration in the 1–4 PRP, not reaching four times that of the peripheral blood. In order to obtain this value, the 1-2 formulation was centrifuged, and the pelleted platelets were resuspended in the adjusted volume according to theoretical calculations. The platelet aggregation was probably induced as a consequence of the centrifugation process. The aggregates would incorrectly be considered as a single platelet by the hematology analyzer, resulting in the low counts found for the 1-4 formulation.

### 3.2. Characterization of the Supernatant Preparations

**Figure 2:**
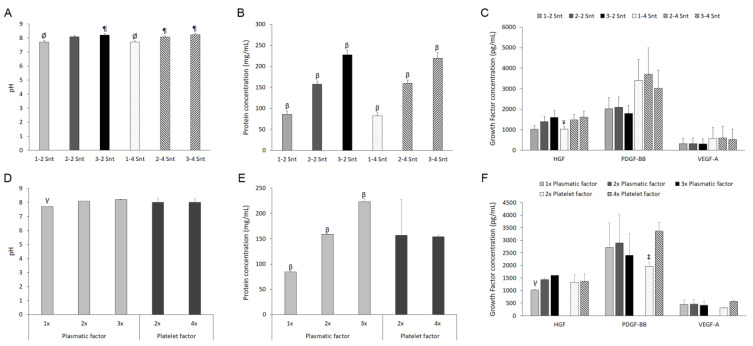
Characterization of the six supernatants obtained from formulations with different plasma and platelet contents. Values of pH (**A**), protein concentration (**B**), and growth factors concentrations (**C**) for the six preparations are graphically represented. Moreover, the effects of both the plasmatic and the platelet factors on these parameters were individually analyzed (**D**–**F**). 1-2 Snt: supernatant with the same plasma content (one times the plasma) and twice the platelet content of the peripheral blood (two times the platelets). 2-2 Snt: supernatant with twice the platelet and plasma contents of the peripheral blood. 3-2 Snt: supernatant with three- and two-times higher concentrations of plasma and platelet contents, respectively, than the peripheral blood. 1-4 Snt: supernatant with the same plasma content and a platelet concentration four times higher than the peripheral blood. 2-4 Snt: supernatant with twice the plasma content and a four-times higher concentration of platelet-derived factors, with respect to the blood. 3-4 Snt: supernatant with three and four times the concentrations of plasma and platelets, respectively, compared to the peripheral blood. *ØStatistically significant differences with respect to the 2-4 Snt, 3-2 Snt, and 3-4 Snt groups. βStatistically significant differences with respect to the supernatants derived from formulations with different plasmatic factors. ¶Statistically significant differences with respect to the 1-2 Snt and 1-4 Snt groups. γStatistically significant differences with respect to the two times and three times plasmatic factor groups. ‡Statistically significant differences with respect to the four times platelet factor group. ¥Statistically significant differences with respect to the 3-4 Snt group (p ≤ 0.05) (n = 5)*.

The 1-0 formulation with one times the plasmatic factor and without any platelets was lyophilized and maintained at 4 °C. The following day, it was reconstituted in different volumes of the 1-2 and 1-4 formulations to obtain the 2-2, 3-2, 2-4, and 3-4 supernatants. Moreover, the 1-2 and 1-4 PRPs were activated in order to prepare the corresponding 1-2 and 1-4 supernatants. All the cell culture supplements were characterized regarding the pH value, total protein content, and growth factor concentration. With respect to the pH, some differences were observed among the six supernatants (Figure 2A), not being associated with the platelet factor but with the plasmatic one (Figure 2D). In this sense, statistically significant lower values were found in the supernatants with a plasma content similar to the peripheral blood than in those with two times and three times the plasmatic factor (7.7 ± 0.0, 8.1 ± 0.0, and 8.2 ± 0.0 for one times, two times, and three times the plasmatic factor content, respectively).

Concerning the total protein content, a statistically significant increase was found to be directly associated with a raised plasma component in the supernatant, regardless of the platelet concentration, with the supplements with a three times plasmatic factor those with the highest protein concentrations (227 ± 12 and 219 ± 13 mg/mL for 3-2 Snt and 3-4 Snt, respectively) (Figure 2B). Taking into account the plasma content individually, the protein concentrations of 84 ± 2, 159 ± 2, and 223 ± 5 mg/mL were measured for the supernatants with the same, two times, and three times the plasma component concentrations of the peripheral blood, respectively (Figure 2E). However, no variation in the total protein content was detected considering only the platelet factor (157 ± 71 and 154 ± 4 mg/mL for supernatants with two times and four times the platelet concentrations of the blood, respectively).

The concentrations of several growth factors were measured in the six supernatants (Figure 2C). No statistically significant differences among the formulations were found regarding the PDGF-BB and VEGF-A molecules. However, a significantly lower HGF concentration was determined in the 1-4 supernatant compared to the formulation with a three times higher plasma concentration and a similar platelet-derived component (3-4 Snt). Figure 2F shows the effect on the growth factor contents of increasing concentrations of plasma and platelet factors when considered separately. The statistically significant differences for the HGF concentration can be clearly attributed to the dissimilar plasma content. Thus, the lowest HGF content was found to be associated with the supernatant with one times the plasmatic factor, and the differences were statistically significant with respect to two times and three times the plasmatic factors (1025 ± 13 vs. 1432 ± 64 and 1605 ± 2, for one times vs. two times and one times vs. three times, respectively). On the contrary, no differences for the PDGF-BB and VEGF-A concentrations were observed regarding the concentration of the plasma components in the supernatant. Concerning the platelet concentration, significative differences were detected for the PDGF-BB. Thus, the content of this growth factor was significatively lower in the supernatants with minor platelet factors (1996 ±160 vs. 3375 ± 345 pg/mL for two times vs. four times the platelet factor). Moreover, a higher VEGF-A concentration was detected in the supernatants with four times the platelet factor with respect to those with half of the platelet concentration, despite not being statistically significant (572 ± 36 vs. 312 ± 10 pg/mL for four times vs. two times, respectively). Finally, no modification in the HGF concentration was promoted by the duplication of the platelet content.

### 3.3. Cell Proliferation Assay

**Figure 3:**
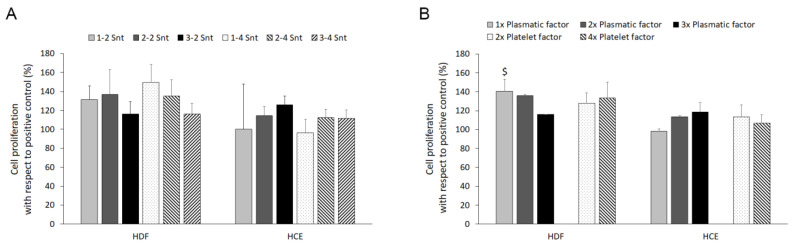
Cell proliferation analysis. Cells from two phenotypes were treated with supernatants (Snts) derived from six PRP-derived formulations. The code of these supernatants consisted of two numbers referring to the plasma and platelet contents, respectively, compared to the peripheral blood. A double statistical analysis was performed to determine the effect of these supernatants on the proliferation rate induced by the specific composition of both plasma and platelet-derived factors (**A**), and, in addition, that induced by the plasma- or platelet-derived factors considered separately (**B**). The results are expressed as a percentage of the proliferation achieved by the cells maintained with the routine culture medium specific for each cell phenotype (the positive control). HDFs: human dermal fibroblasts; and HCE-1: human corneal epithelial cell. *$Statistically significant differences with respect to the three times plasmatic factor group* (*p ≤ 0.05*) (*n = 5*).

In order to test the biological effect of the six supernatants, human dermal fibroblasts and epithelial cells were treated with the 2% supernatant preparations in the corresponding culture medium, depending on the cell phenotype. The results are expressed as a percentage relative to the cell proliferation obtained for the positive control. The differences found between the effect of the supernatants with the same plasma component but different platelet contents for both phenotypes were not statistically significant in any case, as shown in Figure 3A.

When the statistical analysis was performed, considering the plasmatic and platelet factors individually, the opposite pattern for the effect of the plasmatic factor increase on the proliferation of both phenotypes was observed. While raising the plasma component of the supernatant promoted a negative response on the dermal fibroblasts, which was statistically significant for the differences found between the supernatants with one times and three times the plasmatic factor (140% ± 13 vs. 116% ± 0 for one times and three times, respectively), the contrary effect was obtained for the corneal epithelial cells (Figure 3B). Similarly, the opposite proliferative responses to the increase in the platelet component of the supernatant were observed in both cell types, but the differences were not significant in any case.

### 3.4. Cell Chemotaxis

**Figure 4:**
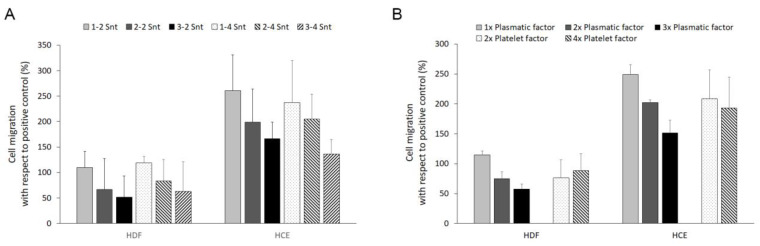
Cell chemotaxis analysis. Cells from two phenotypes were treated with supernatants (Snts) derived from six PRP-derived formulations. The code of these PRPs consisted of two numbers referring to the plasma and platelet contents, respectively, compared to the peripheral blood. A double statistical analysis was performed to determine the effect of these supernatants on the migration rate induced by the specific composition of both plasma- and platelet-derived factors (**A**) and, in addition, that induced by plasma or platelet-derived factors considered individually (**B**). The results are expressed as a percentage of the proliferation achieved by the cells maintained with the routine culture medium specific for each cell phenotype (the positive control). HDFs: human dermal fibroblasts; and HCE-1: human corneal epithelial cell (*n = 5*).

The effects of the six blood-derived supernatants as cell chemoattractants for both the fibroblast and epithelial phenotypes were also tested. The results are expressed as a percentage relative to the cell chemotaxis obtained for the positive control. In no case were observed differences in cell migration attributable to the platelet factor (Figure 4A). While a not very clear cell response to the higher platelet content was found, an inhibitory effect, directly proportional to the increase in the plasma component in the supernatant, was observed for the two cell phenotypes (Figure 4B), although these differences were not statistically significant.

### 3.5. Angiogenesis Assay: Endothelial Cell Proliferation and Tube Formation

**Figure 5:**
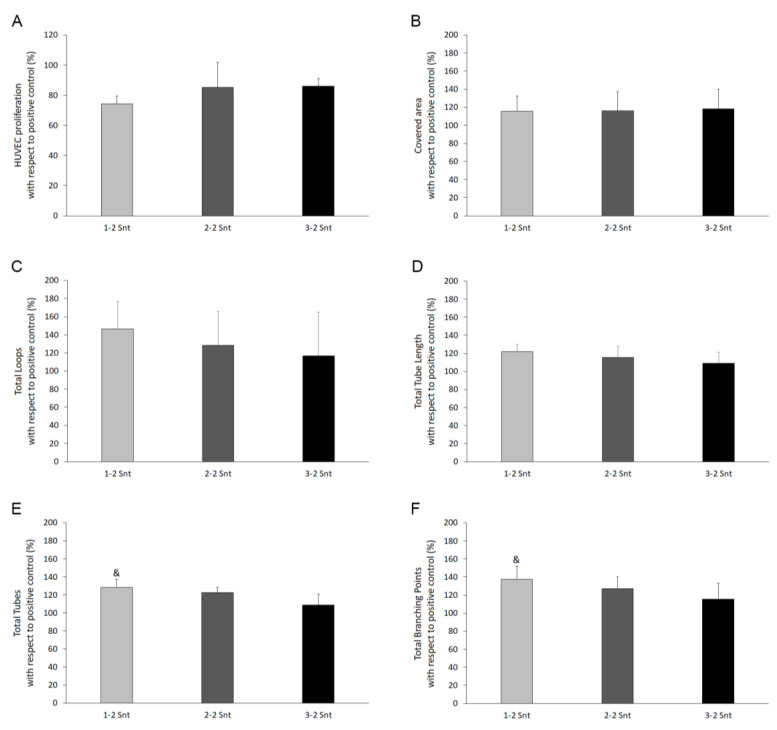
Endothelial cell proliferation analysis and angiogenesis assay. The endothelial cells were treated with supernatants derived from PRP with increasing concentrations of plasmatic factors and setting the platelet content at twice that of the peripheral blood (1-2 Snt, 2-2 Snt, and 3-2 Snt). The proliferation results are expressed as a percentage of the proliferation achieved by the cells under optimal conditions, maintained with the routine culture medium (the positive control) (**A**). For the angiogenesis assay, the endothelial cells were seeded in a µ-plate with 96 wells for a high-throughput 3D cell culture and tube formation assays in which a solubilized basement membrane preparation was previously added. The covered area (**B**), the number of loops (**C**), the total tube length (**D**), the total tubes (**E**), and the total branching points (**F**), considered as the main parameters of the angiogenic process, were measured. The results are expressed as a percentage with respect to the positive control (the routine culture medium). *&Statistically significant differences with respect to the 3-2 Snt group. (p ≤ 0.05).* HUVECs: human umbilical vein endothelial cells *(n = 5)*.

Considering that the supernatants with the highest platelet content did not promote any different cell response, the angiogenic potential was only evaluated for 1-2 Snt, 2-2 Snt, and 3-2 Snt. Thus, the HUVEC proliferation and tube formation ability were assayed after treatment with those three preparations. The DNA concentration value obtained for the cells after the treatment with supernatants was relativized with respect to that of the cells grown under the optimal conditions (the positive control). As can be seen in Figure 5A, the increased concentration of the plasma component in the supernatant did not induce any change in the proliferative rate of the endothelial cells. Regarding the tube formation assay, the parameters such as the covered area, total loops, and total tube length were not affected by increasing the concentration of the plasma content (Figure 5B–D). However, a statistically significant diminished number of total tubes and branching points resulted from the increase in the plasmatic factor in the supernatants (128% ± 9 vs. 109% ± 12 and 138% ± 15 vs. 115% ± 18 for 1-2 Snt vs. 3-2 Snt in the case of the total tubes and branching points, respectively) (Figure 5E,F).

## 4. Discussion

Considering the extreme complexity of the blood plasma component and the fact that diverse roles in key cellular processes have been reported for the extraplatelet biomolecules in an increasing number of studies, the objective of our research was to design and obtain blood-derived formulations with different concentrations of plasma and platelet contents in order to assess their contribution to the biological effect. In this sense, the lyophilization of a platelet-free plasma preparation and its subsequent reconstitution with different volumes of PRP-derived formulations resulted in a range of supernatants with increasing concentrations of plasmatic and platelet-derived factors.

The protein concentration of the different supernatants was in line with the theoretically expected concentration of the plasma component, considering the procedure to obtain it. Thus, the values close to two times and three times the blood plasma protein concentration were found for the 2-2 and 2-4 and for the 3-2 and 3-4 supernatants, respectively. The comparative analysis between formulations differing only in the platelet component (1-2 Snt vs. 1-4 Snt, 2-2 Snt vs. 2-4 Snt, and 3-2 Snt vs. 3-4 Snt) highlighted the practically insignificant platelet contribution to the total protein content. On the other hand, a small but significative rise in the pH values was found in the supernatants with a higher plasmatic factor than the peripheral blood (2-2 Snt, 3-2 Snt, 2-4 Snt, and 3-4 Snt), whereas not in those with a one times plasmatic factor and a higher platelet content (1-4 vs. 1-2). This change might be associated with the lyophilization process, considering that the 1-0 formulation was lyophilized to obtain those supernatants with a higher concentration of the plasma component. The variations in pH could alter the protein configuration and functionality [53,54]; however, previous studies to examine the stability of the 1-2 formulation after lyophilization were performed. Thus, the preservation of the biological properties of this supernatant for up to 3 months was reported [55,56].

Concerning the growth factor content, the supernatants with two and three times the plasma component of the peripheral blood were significantly enriched in HGFs, as this protein circulates freely in the plasma, unlike the other two biomolecules analyzed, which are mainly released by platelets after their activation [57,58,59]. Thus, statistically significant higher PDGF-BB concentrations were found in the supernatants with a higher platelet-derived content, as described in previous research [60]. Regarding the VEGF, in four out of the five donors, the concentration of this growth factor was raised with the increase in the platelet component. However, the differences were not statistically significant, possibly due to the great interindividual variability for the VEGF-A content, as its values ranged from 72 to 631 pg/mL in supernatants with two times the platelet content, as well as from 85 to 1306 pg/mL in those with four times. Therefore, the inclusion of a larger number of donors would probably lead to the achievement of statistical significance.

In order to assess the biological effect of the supernatants with a different balance between the plasma and platelet components, the proliferative and migratory capacities of two cell phenotypes were quantified after treatment with them. In general terms, a higher level of chemotaxis towards the PRP-derived supernatants was found for the corneal epithelial cells with respect to the dermal fibroblasts, whereas a similar proliferative response was shown by both phenotypes. Furthermore, no statistically significant differences were found regarding the potential of the six formulations for promoting proliferation and migration in both cell phenotypes. A possible explanation for the absence of beneficial effects could be the altered balance between the platelet- and plasma-derived negative and positive effectors with respect to the platelet concentrates. Human serum albumin is the most abundant protein in blood plasma, constituting about half of the serum proteins. Despite its theoretically beneficious role, due to its structural and biochemical features [61,62,63,64], there is currently insufficient knowledge on the effect of an elevated concentration on cell behavior as diverse modifications affecting its own function have been described [65]. In addition, the dysregulation of the concentration of many other biomolecules circulating in human blood could induce undesired effects on cells treated with them. This might be the case for some simple lipids such as fatty acids, recently suggested as key regulators of cell survival and proliferation processes [66,67,68,69], or some miRNAs, whose role as the modulators of proliferative and cell cycle pathways still needs to be better elucidated [70].

Another probable reason for these supernatants not inducing especially favorable cellular responses could be the sub-optimal reconstitution of lyophilized preparations with a high concentration of plasma components. This circumstance might have induced conformational changes, inactivation, or inaccurate protein concentrations, promoted by the generation of aggregates in certain cases [71,72]. The HGF concentrations of 1.4 and 1.6 times that of the blood plasma were found for the supernatants with two and three times the plasma content of the blood, respectively, which is probably associated with solubility problems. In this sense, the prevention of protein aggregation in lyophilized human plasma has been reported when freeze-drying is performed in the presence of sugars such as trehalose, which would protect it over a wide range of temperatures [73].

The previously described effects on cell proliferation and migration were found regardless of the platelet content concentration in the supernatant. For this reason, it was decided to perform the angiogenesis assays including the formulations in which the plasma content differed, but the concentration of the platelet component remained constant at two times. No differences in the proliferation-inducing effect were found when comparing the supernatants whose plasma content was increased. However, the endothelial cell ability to develop the blood tube network was significantly reduced after treatment with the supernatant in which the plasma content was three-fold higher than in the peripheral blood. This antiangiogenic effect, together with the fact that the increasing concentrations of the plasma components do not significantly impair the potential to promote cell proliferation and migration, makes these formulations a very promising approach for therapies, where the deleterious consequences of neovascularization should be avoided [74,75].

## 5. Conclusions

In summary, new blood-derived preparations with different concentrations of plasmatic and platelet factors have been developed in this current research, and their biological effect on several cell phenotypes has been reported. Considering that the number of donors included for testing the PRP formulations could be a limitation of this present research, the results have shown that increasing the plasma component has not implemented any variation in the potential of the PRP-derived formulations. Therefore, the 1-2 formulation corresponding to PRGF would be the optimal PRP, due to its regenerative potential, as well as the simpler protocol for obtaining it. The identification of plasma components, such as specific electrolytes, whose concentration is increased, that possibly counteract the platelet stimulatory effect could be considered for further research. However, the preparations with a higher concentration of plasma content have emerged as promising new approaches to enhance tissue regeneration, avoiding neovascularization, which is required in the treatment of certain diseases.

## Figures and Tables

**Table 1:** Hematologic characterization of the blood-derived plasma formulations and the blood from which they were obtained. ERY: erythrocytes; PLA: platelets; LEU: leucocytes; PLA ENR: platelet enrichment with respect to blood; and N.A.: not applicable (*n = 5*).

Parameter	Blood	Formulations		
		1-0	1-2	1-4
ERY (×10^6^/μL)	4.5 ± 0.5	0.0 ± 0.0	0.0 ± 0.0	0.0 ± 0.0
LEU (×10^3^/μL)	5.5 ± 1.2	0.0 ± 0.0	0.2 ± 0.1	0.4 ± 0.1
PLA (×10^3^/μL)	221 ± 41	0.0 ± 0.0	390 ± 58	722 ± 124
PLA ENR (Formulation/blood)	N.A.	N.A.	1.9 ± 0.2	3.3 ± 0.6

## Data Availability

The raw data supporting the conclusions of this article will be made available by the authors on request.
